# Overexpression of MicroRNA-148b-3p stimulates osteogenesis of human bone marrow-derived mesenchymal stem cells: the role of MicroRNA-148b-3p in osteogenesis

**DOI:** 10.1186/s12881-019-0854-3

**Published:** 2019-07-01

**Authors:** Samaneh Mollazadeh, Bibi Sedigheh Fazly Bazzaz, Vajiheh Neshati, Antoine A. F. de Vries, Hojjat Naderi-Meshkin, Majid Mojarad, Mahdi Mirahmadi, Zeinab Neshati, Mohammad Amin Kerachian

**Affiliations:** 1 Natural Products and Medicinal Plants Research Center, North Khorasan University of Medical Sciences, Bojnurd, Iran; 2 Biotechnology Research Center, Pharmaceutical Technology Institute, Mashhad University of Medical Sciences, Mashhad, Iran; 3 Department of Food and Drug Control, School of Pharmacy, Mashhad University of Medical Sciences, Mashhad, Iran; 4 School of Pharmacy, Mashhad University of Medical Sciences, Mashhad, Iran; 5 Department of Cardiology, Leiden University Medical Center, Leiden, the Netherlands; 6 Stem Cell and Regenerative Medicine Research Group, Academic Center for Education, Culture Research (ACECR), Khorasan Razavi Branch, Mashhad, Iran; 7 Medical Genetics Research Center, Mashhad University of Medical Sciences, Mashhad, Iran; 8 Department of Medical Genetics, Faculty of Medicine, Mashhad University of Medical Sciences, Mashhad, Iran; 9 Department of Biology, Faculty of Science, Ferdowsi University of Mashhad, Mashhad, Iran

**Keywords:** Mesenchymal stem cells, Lentivirus, MicroRNA-148b, Osteogenesis

## Abstract

**Background:**

Mesenchymal stem cells (MSCs) are attractive choices in regenerative medicine and can be genetically modified to obtain better results in therapeutics. Bone development and metabolism are controlled by various factors including microRNAs (miRs) interference, which are small non-coding endogenous RNAs.

**Methods:**

In the current study, the effects of forced miR-148b expression was evaluated on osteogenic activity. Human bone marrow-derived mesenchymal stem cells (BM-MSCs) were transduced with bicistronic lentiviral vector encoding hsa-miR-148b-3p or -5p and the enhanced green fluorescent protein. Fourteen days post-transduction, immunostaining as well as Western blotting were used to analyze osteogenesis.

**Results:**

Overexpression of miR-148b-3p increased the osteogenic differentiation of human BM-MSCs as demonstrated by anenhancement of mineralized nodular formation and an increase in the levels of osteoblastic differentiation biomarkers, alkaline phosphatase and collagen type I.

**Conclusions:**

Since lentivirally overexpressed miR-148b-3p increased osteogenic differentiation capability of BM-MSCs, this miR could be applied as a therapeutic modulator to optimize bone function.

**Electronic supplementary material:**

The online version of this article (10.1186/s12881-019-0854-3) contains supplementary material, which is available to authorized users.

## Background

Human bone marrow-derived mesenchymal stem cells (BM-MSCs) are multipotent cells, which can differentiate into various types of specialized mesodermal cells including osteoblasts. MSCs are attractive candidates for regenerative medicine applications due to ease with which they can obtained, their growth properties and their differentiation potential [1, 2]. However, effective osteogenic differentiation of MSCs has become an important issue in tissue engineering [3]. In this regard, genetic modification of MSCs with microRNAs (miRs) could be a promising approach to improve osteogenesis in certain skeletal diseases [4, 5].

MiRs are abundant class of small (~ 22-nucleotide) single-strand and non-coding regulatory RNA molecules that bind to target mRNAs in a sequence-specific manner mostly resulting in the translation repression or mRNA degradation [6]. MiRs have been shown to participate in a wide range of physiological and pathological process [7] including osteoblastogenesis [6, 8]. While miRs such as miR-26a, miR-26b, miR-29b [9], miR-148b [10], miR-335-5p [11], and miR-378 [12] enhance osteogenesis, other miRs including miR-31 [13], miR-125b [14], and miR-433 [15] negatively affect bone formation.

Important issues regarding the therapeutic application of miRs are the specificity and duration of their effects. Viral delivery of short hairpain (sh) RNAs corresponding to mature miRNA into cells is an efficient mean to impose miR-mediated control of gene expression [16]. Lentiviral vectors (LVs) are widely applied in fundamental research and now they are also entering the clinic due to their ability to accomplish stable expression of transgenes in dividing and non-dividing cells [17, 18]. Besides for exogenous expression of protein-encoding genes, LV can also be used to modulate expression of genes via shRNA-mediated RNA interference both in vitro and in vivo [18, 19]. To study the individual roles of two mature miRs derived from miR-148b (i.e. miR-148b-3p and miR-148b-5p) on osteoblastogenesis, shRNAs corresponding to miR-148b-3p and miR-148b-5p were overexpressed in human BM-MSCs using a vesicular stomatitis virus G protein-pseudotyped human immunodeficiency virus type 1 (HIV1)-based LV.

## Methods

### Isolation and characterization of human BM-MSCs

Bone marrow aspirates of healthy donors undergoing therapeutic surgery were used to isolate MSCs as previously described [20]. The entire procedure was approved by the Ethics Committee of Mashhad University of Medical Sciences and carried out with written informed consent of the donors. Briefly, bone marrow samples were aspirated in a sterile condition during surgery. Then, they were aliquoted into 75-cm^2^ culture flask by adding Dulbecco’s modified Eagle’s medium-low glucose (DMEM-LG) (Gibco, Paisley, Scotland) consisting 20% fetal bovine serum (FBS; Gibco, Paisley, Scotland) as well as 1× penicillin/streptomycin (Pen/Strep; Gibco, Paisley, Scotland). Cell suspension was maintained at 37 °C in a humidified incubator with 5% CO_2_. After 3 days, cultures were rinsed with 1× phosphate-buffered saline (PBS) twice to eliminate non-adherent cells and allowed to reach confluency. Then, confluent cells were trypsinized and subcultured at a density of 5–10 × 10^3^ cells/cm^2^ in new flasks. The characterized BM-MSCs in terms of cell morphology, cell markers and osteogenic and adipogenic capability were used for this study at passage three.

### Design the miRNA sequences and plasmid construction

**Fig. 1:**
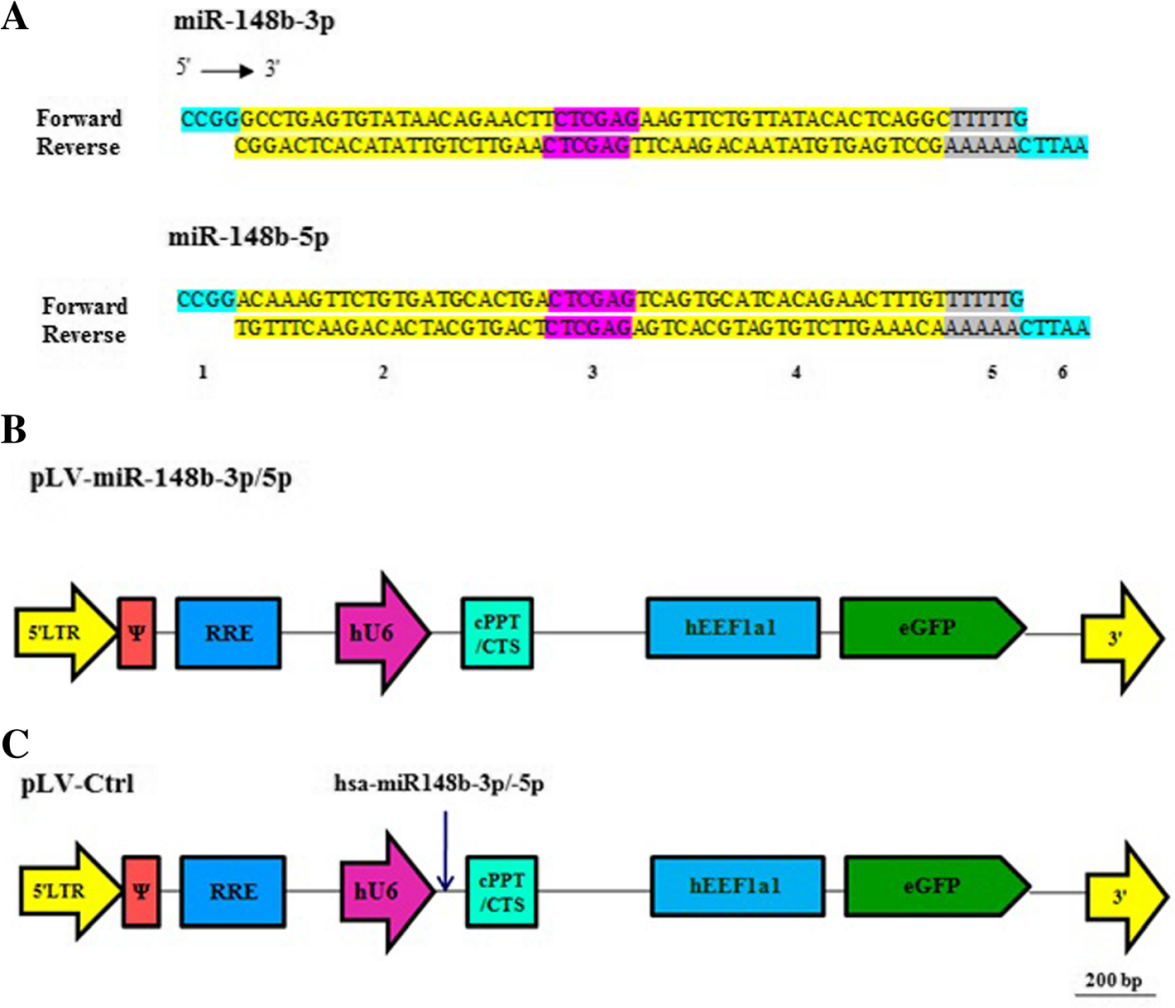
Cassettes and structure of LV shuttle plasmids. **a** Schematic representation of miR-148b-3p and miR-148b-5p sequences after ligation of forward and reverse primers. Section 1 and 6 are SgrA1 and EcoRI restriction sites, respectively. Section 2 and 4 are sense and antisense strands, respectively. Section 3 is necessary for hairpin structure (loop). Section 5 is a terminator (poly T). **b** Maps of pLV-miR-148b-3p and pLV-miR-148b-5p (**c**) and of pLV-Ctrl.5′ LTR, chimeric 5′ long terminal repeat including the Rous sarcoma virus U3 region as well as the HIV1 R and U5 regions; Ψ, HIV1 packaging signal; RRE, HIV1 Rev-responsive element; hU6, human U6 gene promoter; miR-158b-3p/5p, coding sequence of desired miRNAs; cPPT/CTS, HIV1 central polypurine tract and termination site; hEEF1a1, eukaryotic translation elongation factor 1 alpha 1 gene promoter; EGFP; enhanced green fluorescent coding sequence; 3′ LTR, 3′ HIV1 long terminal repeat involving a deletion in the U3 region to render the LV self-inactivating

Naturally, primary miRNAs are produced after transcription of miRNAs by RNA polymerase II. Then, primary miRNAs are cleaved to hairpin pre-miRNAs which are transferred to cytoplasm followed by processing into RNA duplex (~ 22-nucleotide) [21]. The mechanism of RNA splicing can be more efficient when pre-miRNA are flanked by specific sequences [22, 23]. In an attempt to improve our expression strategy, we designed our miRNA based on the pattern as indicated below. The sequences of the mature hsa-miR-148b-3p (5′-UCAGUGCAUCACAGAACUUUGU-3′) as well as hsa-miR148b-5p (5′-AAGUUCUGUUAUACACUCAGGC-3′) were obtained from miRBase (http://www.mirbase.org/). Besides, anti-mature sequences were designed as complementary sequences of matures. According to this, oligonucleotides pairs (forward and reversed) were annealed for both miRNAs. After ligation, the whole sequences were flanked by XhoI recognition site, which is essential for constructing hairpin structures. Also, the two restriction sites were developed at the both 5′ and 3′ ending sites followed by a sequences of 5 T residues (RNA polymerase III transcription termination signal) (Fig. 1a). Then, hybridized products of these oligonucleotids and their reverse stretches were cloned in between the unique SgrAI and EcoRI recognition site of the LV shuttle construct SHC007-hEEF1a1.EGFP. SHC007-hEEF1a1.EGFP is derived from the shRNA plasmid DNA control vectors SHC007 (Sigma-Aldrich, St. Louis, MO), in which the human phosphoglycerate kinase 1 gene promoter as well as the *Streptomyces alboniger* puromycin N-acetyl-transferase-coding sequence are replaced by the human eukaryotic translation elongation factor 1 alpha 1 gene promoter and the coding sequence of the enhanced green fluorescent protein (EGFP) of *Aequorea Victoria*. The consequential plasmids, pLV.hU6.miR-148b-3p.hEEF1a1.EGFP (hereinafter called pLV-miR-148b-3p) and pLV.hU6.miR-148b-5p.hEEF1a1.EGFP (hereinafter referred to as pLV-miR-148b-5p) interceded the expression of miR-148b-3p and miR-148b-5p, respectively. Both plasmids also encode the EGFP expression. The correctness of DNA constructs were approved by restriction endonuclease digestions (using *AccI*, *HaeII*, *PVuI*, and *XhoI*) as well as nucleotide sequence analysis. Sequencing was accomplished through Leiden Genome Technology Center (http://www.lgtc.nl/) using a BigDye Terminator v3.1 Cycle Sequencing Kit and a 3730xl DNA Analyzer (both from Thermo Fisher Scientific, Waltham, MA). Enzymes used in this study were purchased from New England Biolabs (BIOKÉ, Leiden, the Netherlands) and Fermentas (Thermo Fisher Scientific, USA) and applied according to the standard protocols. Detailed genetic maps of pLV-miR-148b-3p, pLV-miR-148b-5p as well as SHC007-hEEF1a1.EGFP (hereinafter designated pLV-Ctrl) are provided in Fig. 1b, c.

### Lentiviral vectors production

To construct lentiviral vector particles, HEK (293 T) cells were infected with transfection medium composed of molar ratio of 1:1:2 packaging plasmids [PSPAX2 (Addgene; plasmid number; 12,260), pLP/VSVG (Life Technologies Europe)] and lentiviral vector shuttle plasmid pLV-miR-14bb-3p/miR-148b-5p (to generate LV-miR-148b3p/−5p particles) or pLV-Ctrl (to produce control vector [LV-Ctrl] particles) supplemented with NaCl 150 mM, and 25-kDa polyethylenimine (PEI; PolysciencesEurope, Hirschberg an der Bergstrasse, Germany). Following overnight incubation of DNA–PEI complexes, medium was refreshed with DMEM-LG supplemented with 5% FBS and 25 mM HEPES-NaOH (pH 7.4). After 48 h, supernatants of transfected cells were collected and centrifuged to remove cellular debris followed by filtering through 0.45-μm pore-sized, 33-mm diameter polyethersulfone Millex-HP syringe filters (Millipore, Amsterdam, the Netherlands). Viral particles were precipitated by adding a cushion of 20% (wt/vol) sucrose to the cleared supernatants, and then ultracentrifuged at 15000 rpm for 2 h at 4 °C in a SW32 swinging bucket rotor (Beckman Coulter Nederland, Woerden, the Netherlands). Finally, the supernatants were thoroughly removed and PBS (1%) containing 1% bovine serum albumin (Sigma-Aldrich Chemie, Taufkirchen, Germany) were added to viral pellet followed by gentle rocking overnight on the shaker (110 rpm) at 4 °C. As a final point, viral vectors were aliquoted in 1.5 ml microtubes and stocked at − 80 °C.

### Lentiviral transduction

Human BM-MSCs were seeded at density of 5 × 10^3^ cells/cm^2^. The following day, the cells were incubated for ±20 h with LV-miR-148b-3p or LV-miR-148b-5p or LV-Ctrl in culture medium supplemented with 2.5 μg/ml DEAE-dextran sulfate (Sigma-Aldrich Chemie, Taufkirchen, Germany). The other day, cells were rinsed with PBS and the transfection medium was changed with osteogenic differentiation medium (ODM) containing DMEM-LG included 15% FBS, and 100 U/ml Pen/Strep supplemented with 10 mM ß-glycerophosphate, 50 μg/ml ascorbic acid, and 0.1 μM dexamethasone (all from Sigma-Aldrich Chemie, Taufkirchen, Germany). To check the transduction efficiency, direct EGFP fluorescence was evaluated at day 7 using an inverse fluorescence microscopy (Nikon Eclipse TE2000-U, Tokyo, Japan).

### Mineralization assay

Twenty-one days after osteogenic induction in order to stain calcified nodules, cells were washed twice with PBS and fixed with 10% formalin (Sigma-Aldrich Chemie, Taufkirchen, Germany) for 45 min at room temperature (RT). Then, fixed cells were incubated with 1% Alizarin red S staining solution [ARS; Sigma-Aldrich Chemie (pH 4.1–4.3)] for 30 min to develop mineralized bone nodules as previously described [24]. Calcium deposition was identified as orange and red bodies under light microscope (Nikon Eclipse TE2000-U) [25, 26]. To quantify absorbed ARS [27], stained cells were detached using 10% acetic acid (Sigma-Aldrich Chemie, Taufkirchen, Germany) and homogenized. Then, the acidity of the supernatants was neutralized by 10% ammonium hydroxide. Finally, the absorbance was determined at the wavelength of 405 nm by plate reader (BioTek, Bad Friedrichshall, Germany).

### Alkaline phosphatase (ALP) assay

ALP assay was performed to detect intracellular ALP enzyme activity in treated cells. Twenty-one days after osteogenic induction, cells were washed twice with PBS and fixed with 10% formalin, rinsed with PBS, and treated with BCIP/NBT (5-bromo-4-chloro-3-indolyl phosphate/Nitrobluetetrazolium; Sigma-Aldrich Chemie, Taufkirchen, Germany) for 10 min at RT. ALP-positive cultures were stained blue/purple. To quantify ALP activity, differentiated cells were scraped using lysis buffer [50 mM Tris-HCl (pH 7.4) supplemented with 1% Triton X-100 (Sigma-Aldrich Chemie, Taufkirchen, Germany)] and incubated with p-nitro-phenyl phosphate (pNPP; Sigma-Aldrich Chemie, Taufkirchen, Germany) for 30 min at RT. Finally**,** the absorbance of the yellowish product p-nitro-phenol (pNP) was detected spectrophotometrically at 405 nm. The rate of pNP production demonstrates the amounts of ALP produced by differentiated cells.

### Immunostaining

To immunostain markers of interest in human BM-MSCs, they were osteo-inducted at the density of 1 × 10^5^ cells/well in 48-well plates (BD Biosciences, San Jose, CA). Fourteen days after osteogenic differentiation, induced cells were washed twice with PBS and fixed with PBS (1×) containing 4% paraformaldehyde at RT for 30 min. Cells were permeabilized with 0.1% Triton X-100 in PBS and then incubated overnight at 4 °C in the presence of mouse anti-alkaline phosphatase (ALP; BD Biosciences, San Diego, US) and collagen type I (ColI; Sigma-Aldrich Chemie, Taufkirchen, Germany) primary antibodies diluted 1:200 and 1:1000 in PBS + 0.1% donkey serum (Santa Cruz Biotechnology, Santa Cruz, CA), respectively. The next day, cells were incubated with Alexa Fluor 568-conjugated donkey anti-mouse IgG (H + L) secondary antibody (Life Technologies Europe, Bleiswijk, the Netherlands) diluted 1:400 in PBS + 0.1% donkey serum for 4 h at RT. Finally, the nuclei were stained with Hoechst 33258 staining (Sigma-Aldrich Chemie, Taufkirchen, Germany) for 10 min. Cells were completely washed with PBS after each step. Images were taken with a fluorescence microscope attached to digital color camera. The Mean fluorescence signal intensity was determined using ImageJ (version 4.1; National Institutes of Health, Bethesda, MA).

### Western blotting

Two weeks after osteogenesis, induced cells were lysed using Trizol reagent (Invitrogen, Massachusetts, USA) followed by total protein extraction based on the supplier protocol. Then, the concentrations of purified proteins were evaluated by Bradford protein assay kit (Thermo Scientific, Waltham, MA) in which bovine serum albumin (BSA) was used as the standard protein. To determine alkaline phosphatase (ALP) protein expression by Western blotting, 15 μg/ml denatured protein samples were loaded onto sodium dodecyl sulfate (SDS)-containing sample buffer, boiled for 5 min and applied to a 12% SDS-polyacrylamide gel and electrophoresed in Tris-glysine buffer system (Bio-Rad, Hercules, California, USA), which was conducted at 120 V for 60 min. The proteins were then transferred onto polyvinylidene difluoride (PVDF; Bio-Rad, California, USA) membrane in 1× transfer buffer for 1 h at 250 mA in a transblot electrophoretic transfer cell (Bio-Rad California, USA). Membrane was blocked with Tris-buffered saline containing 5% BSA diluted in 0.1% Tween-20 (TBS-T solution) for 3 h at RT and then rinsed with TBS-T solution. For immunodetection of ALP, the blocked membrane was incubated with mouse anti-ALP primary antibody diluted 1:500 in TBS-T plus BSA 2.5% for 1 h at RT, washed four times with TBS-T solution for 20–30 min, and treated with 1:1000 diluted anti-mouse horseradish peroxidase (HRP)-conjugated secondary antibody (Santa Cruz Biotechnology, Texas, USA) for 1 h at RT. Finally, the signals were visualized using chemiluminescence ECL detection kit (Bio-Rad, California, USA) and exposed to X-ray film (Eastman Kodak, Rochester, New York, USA) for 30 s to 10 min. β-actin [mouse anti- β-actin diluted 1:800 (Santa Cruz Biotechnology; Texas, USA)] was used as the standard protein.

### Statistical analysis

All experiments were conducted in triplicates and values were presented as the mean ± standard deviation (SD). The statistical analysis was performed with ANOVA followed by post hoc multiple comparison analysis using Prism 6 (Graphpad Software, San Diego, CA). Differences with *P* values ≤0.05 were considered as statistically significant.

## Results

### Identification of BM-MSCs

Human BM-MSCs could adhere to plastic surface based on their typical properties. The phenotype of primary human BM-MSCs was verified by MSC immunophenotyping, the capacity to form single spindle-fibroblastic cells, and the adipogenesis and osteogenesis potentials. As previously demonstrated, these cells were positive for CD44, CD90, and CD105 (MSC cell surface markers), whilst they were negative for CD34, CD45 (hematopoietic cell markers), and CD11b (pan-macrophage marker) [20]. Alizarin red S staining showed the ability of the primary human BM-MSCs to form mineralized nodules. Moreover, Oil Red O staining displayed that these cells could accumulate lipid droplets under adipogenic conditions. Taken together, findings of osteogenesis and adipogenesis tests displayed that human BM-MSCs have multipotential differentiation properties.

### Production and functional testing of LV particles

One day after transfection of HEK 293 T cells with LV shuttle and packaging plasmids, almost all cells were positive for EGFP, which indicated the transfection efficiency was high. To check the gene transfer activity of the LV-miR-148b-3p, LV-miR-148b-5p, and LV-Ctrl particles, 5 × 10^4^/cm^2^ HEK 293 T indicator cells were treated with 3 μl of a 1000-fold dilution of all three vector stocks. The results of EGFP expression confirmed all vectors were functional with a similar titer. We used empty LVs as a negative control to ignore the probable effects of plasmid backbones in order to study the osteogenic effect of LV-miRs (miR-3p or miR-5p).

### Overexpression of miR-148b-3p promotes osteoblastic differentiation

**Fig. 2:**
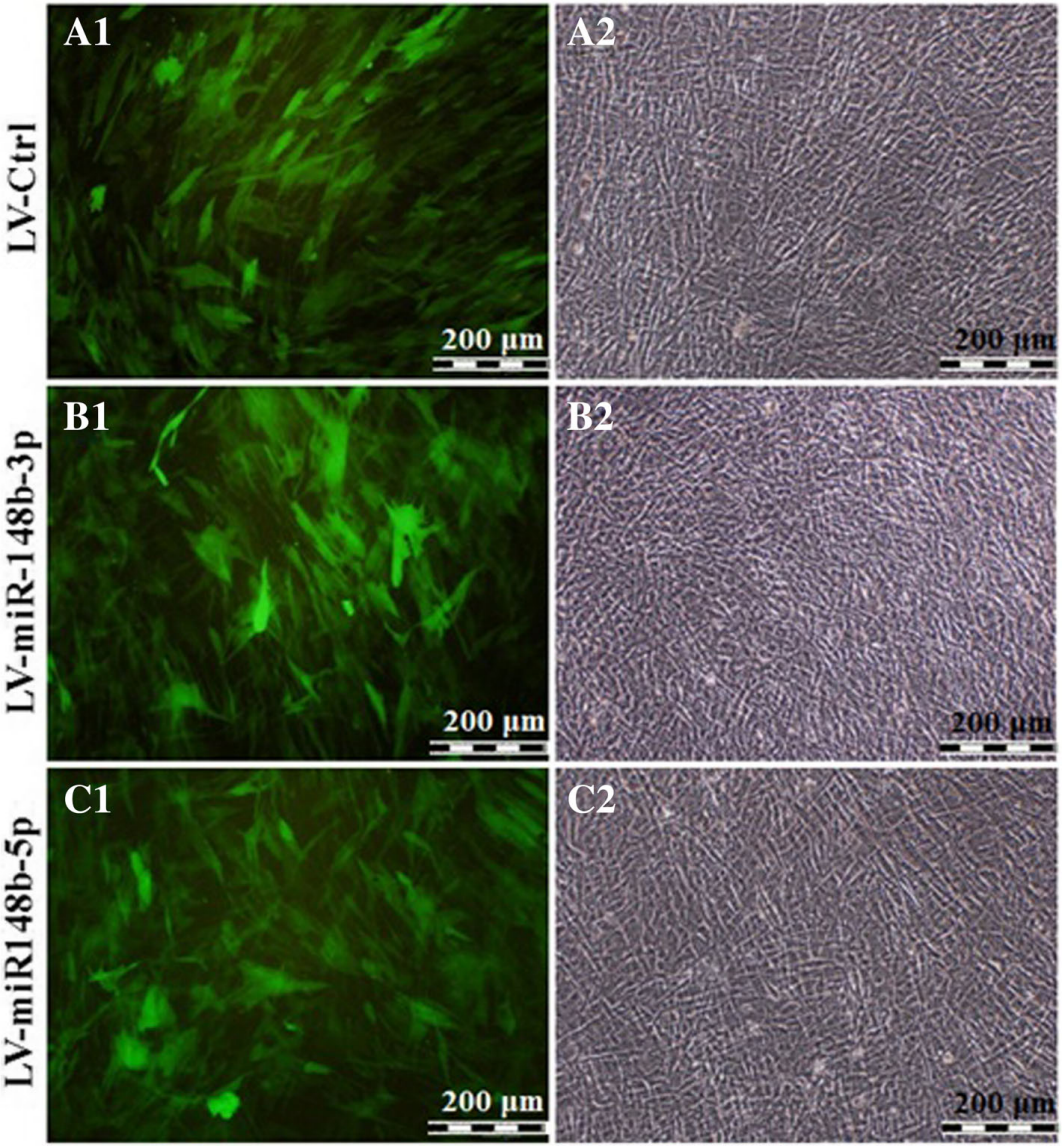
Fluoromicrographs (A1, B1, C1), and bright field (A2, B2, C2) images of LV-Ctrl, LV-miR-148b-3p, and LV-miR-148b-5p-transduced human BM-MSCs that were cultured in osteogenic differentiation medium for 21 days

To investigate the osteogenic effects of permanent expression of hsa-miR-148b, human BM-MSCs were infected with LV-miR-148b-3p, which codes both miR-148b-3p and EGFP, or LV-miR-148b-5p, which express both miR-148b-5p and EGFP or LV-Ctrl, that codes only EGFP. The transduction efficiency of the human BM-MSCs 3 days post-transduction was about 90% as indicated by direct EGFP fluorescence microscopy. Then, transduced cells were incubated in ODM for 21 days. Although, LV functionality were similar in all groups (Fig. 2, left column), the amounts of osteogenic differentiation was significantly more in the cells transduced with LV-miR-148b-3p as shown by white calcified matrix (Fig. 2, right column). In the following, osteo-induction was assayed by alizarin red staining (ARS) as well as alkaline phosphatase (ALP) assay.

**Fig. 3:**
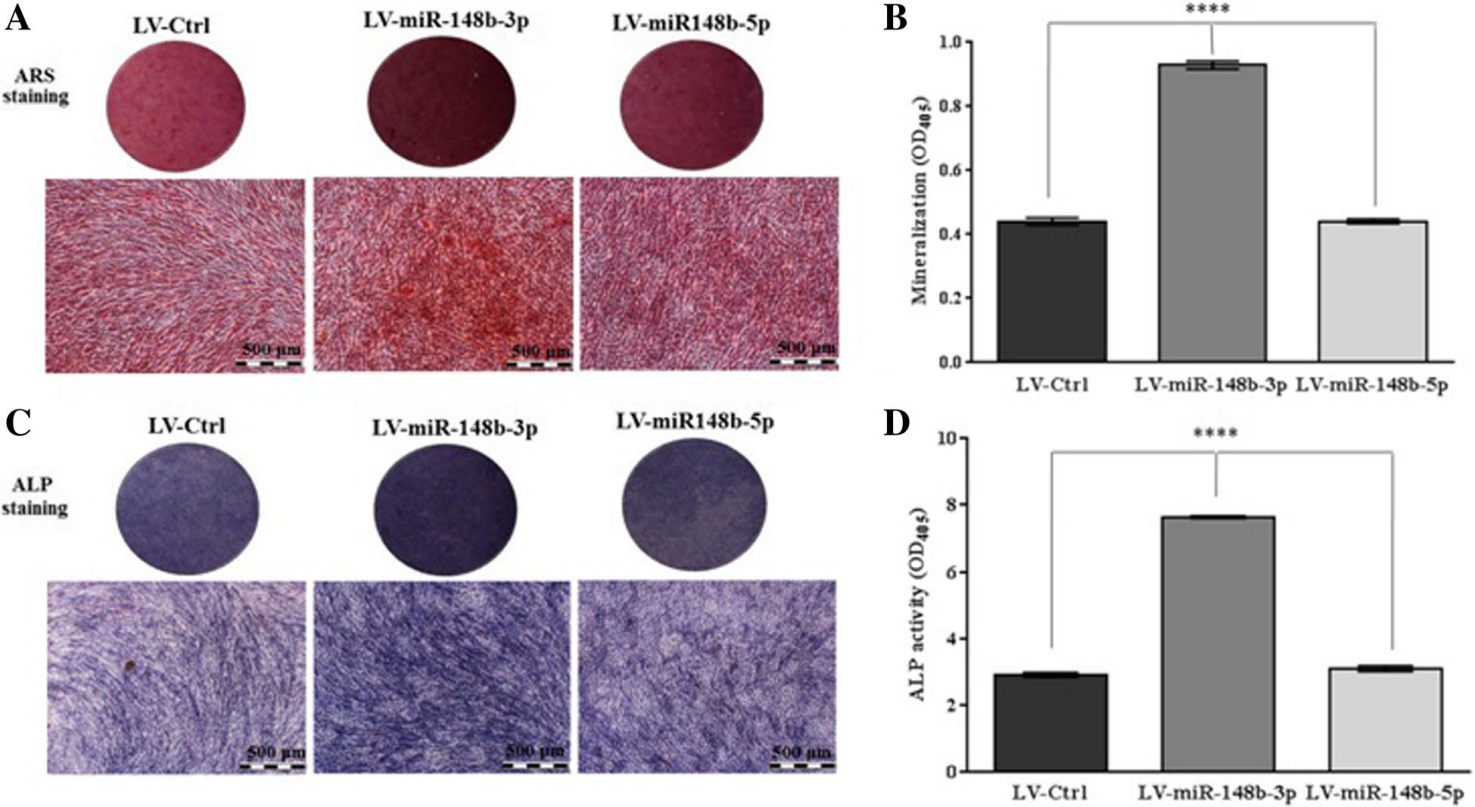
Increased expression of miR-148b-3p in human BM-MSCs was potentially mediated by LV enhanced osteogenic differentiation. **a** After 21 days mineral nodules depositions were detected with ARS staining. **b** The stained cells were eluted using 10% acetic acid and the absorbance of extracted stain was measured at 405 nm to quantify the mineralization level. **c** ALP production was evaluated with BCIP/NBT kit as dark purples deposits. **d** After 14 days ALP activity was quantified with pNPP substrate system.****P < 0.0001

**Fig. 4:**
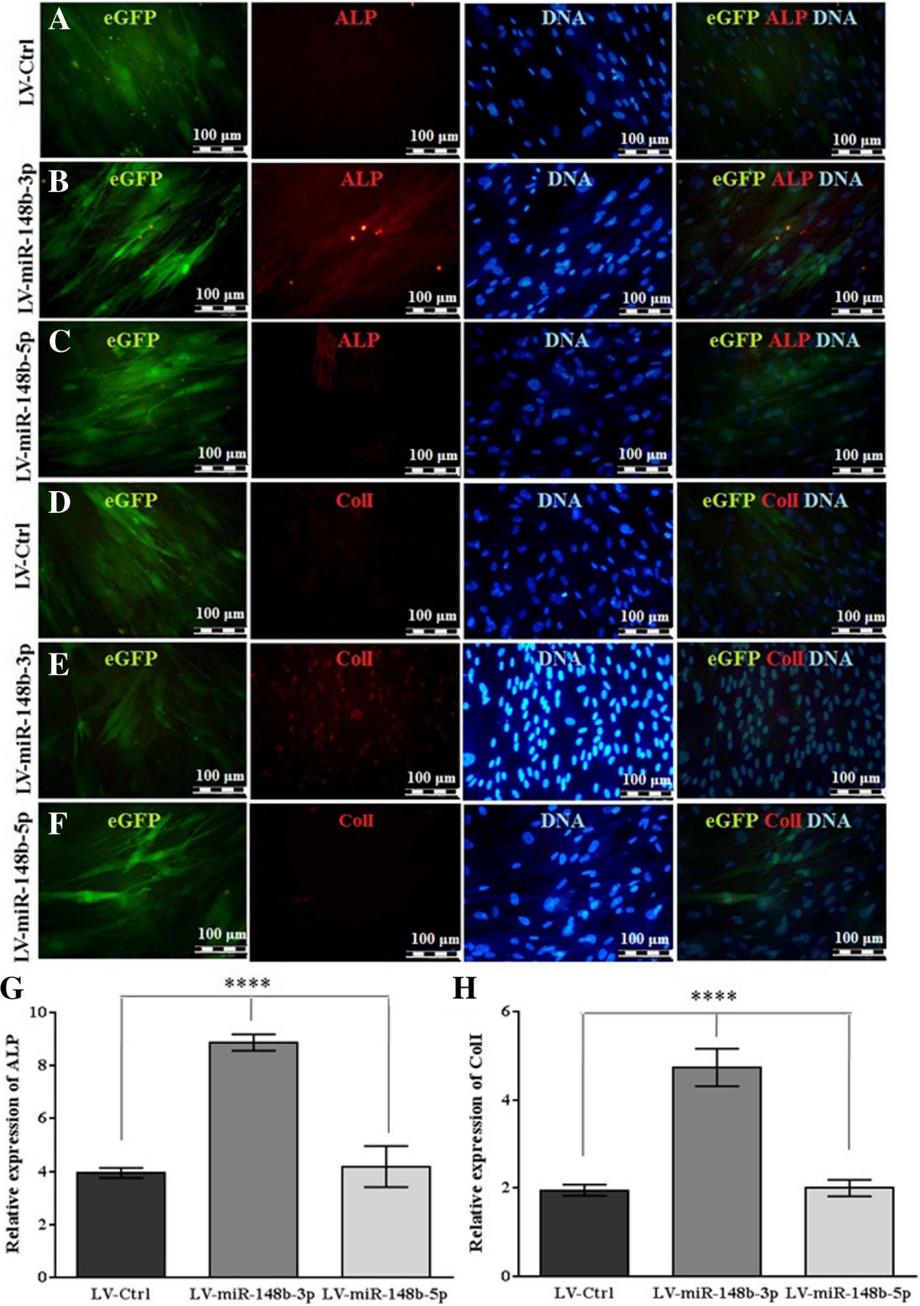
Osteogenic differentiation of human BM-MSCs was increased by miR-148b-3p overexpression. (**a**-**f**) Representative micrograph of 14-day incubation of human BM-MSCs exposed to LV-Ctrl, LV-miR-148b-3p or LV-miR-148b-5p. Immunostaining of ALP and ColI with nuclear counterstained (Hoechst) were shown in red and blue respectively. It should be mentioned that EGFP+ cells were in green. (**g**-**h**)BM-MSCs overexpressed miR-148b-3p demonstrated 2.2- and 2.5-fold enhancement in ALP and ColI expression respectively compared to other groups. ****shows significant differences (*P* < 0.0001) between LV-miR-148b-3pwith LV-miR-148b-5p and LV-Ctrl

As demonstrated in Fig. 3a (Additional file 1 Figure S1), miR-148b-3p overexpression resulted in more bright red bone nodular formation in human BM-MSCs in a time dependent manner. Consistently, ARS quantification displayed 1.5-fold increase in calcification in LV-miR-148b-3p-transduced cells in comparison with LV-miR-148b-5p- and LV-Ctrl-transduced cells (Fig. 3b). Similar data was achieved from ALP staining (Fig. 3c) in which miR-148b-3p-overexpressed human BM-MSCs revealed dark purple staining and 2.5-fold enhancement in ALP production compared to LV-miR-148b-5p- and LV-Ctrl-transduced cells (Fig. 3d). Also, the results of immunostaining (Fig. 4) implicated that expression of miR-148b-3p in human BM-MSCs resulted in an enhanced expression of about 2.5-fold in ALP and ColI compared to LV-miR-148b-5p- and LV-Ctrl–transduced cells.

**Fig. 5:**
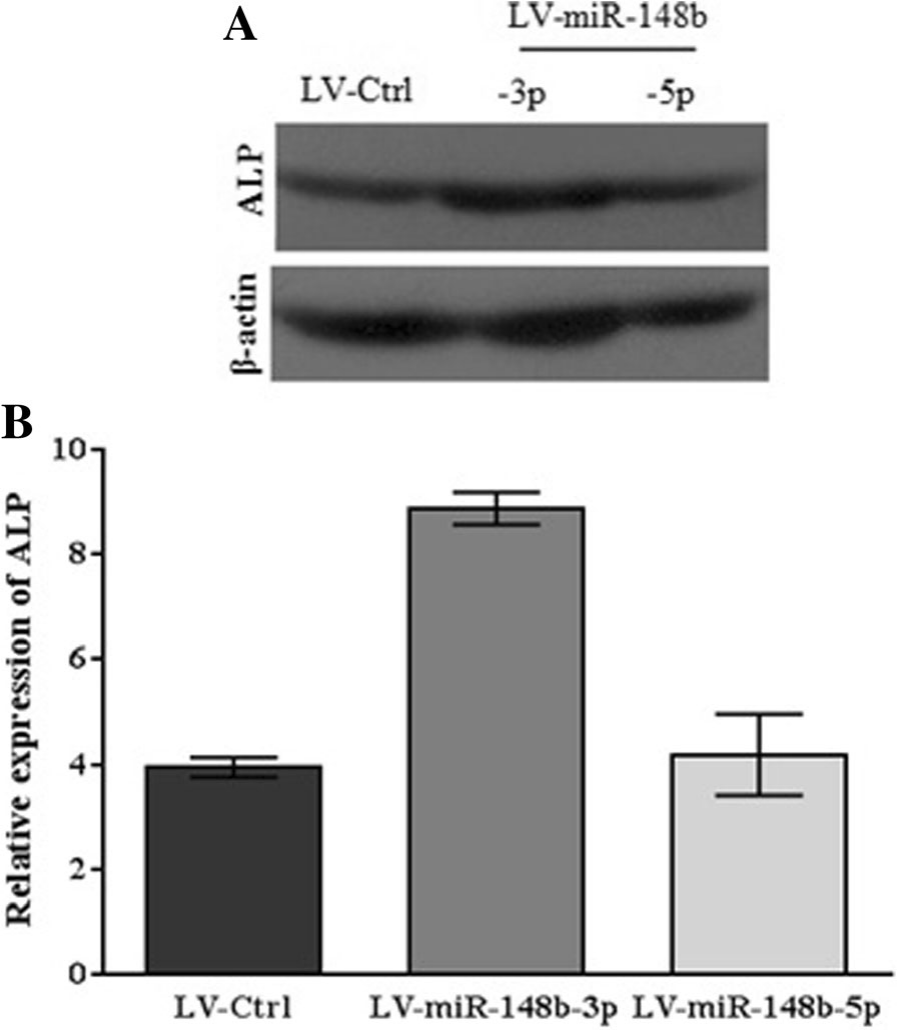
The exogenous overexpression of miR-148b-3p upregulated protein expression level of ALP. **a** 14 days post-transduction, ALP were expressed by the cells in all infected groups (LV-Ctrl, LV-miR-148b-3p, LV-miR-148b-5p). In contrast, ALP exhibited greater expressionin the cells treated with miR-148b-3p. The resultswere shown by the band density ratio of ALP with β-actin. **b** The relative expression of ALP (fold change) after quantification of Western blotting analysis

Western blot analysis, on day 14th of post differentiation displayed immunoreactive bands with a weight of 89 kDa in response to the anti-ALP antibody (Fig. 5). Although Western blot analysis revealed that ALP was expressed in all three groups, LV-miR-148b-3p-treated cells exhibited 1.2-fold enhancement in ALP expression compared to LV-miR-148b-5p-transduced cells. Altogether, these data demonstrated stimulatory effects of miR-148b-3p overexpression on osteogenic differentiation in human BM-MSCs.

## Discussion

Several studies have shown that BM-MSCs can be genetically modified and applied in local or systemic therapies [28]. Nevertheless, understanding the multistep process of osteogenesis allow us to develop therapeutic intervention in skeletal problems/complications [29]. Skeletogenesis is tightly regulated through numerous molecular players and cellular mechanisms. Furthermore, the balance between the different bone cells is critical for bone maintenance [30]. There is expanding evidence demonstrating that miRs can affect osteogenic differentiation through inhibiting protein synthesis of their targets [31]. The miR profiling strategy revealed that miR-148b positively regulates osteogenic cell fate [10]. Thus, we aimed to elucidate the overexpression of miR-148b arms (−3p or -5p) on osteogenesis. To do so, we introduced LVs carrying hsa-miR-148b-3p or -5p into human BM-MSCs.

Our data strongly pointed that miR-148b-3p could increase osteogenic differentiation in human BM-MSCs as demonstrated by ARS and ALP staining. Consistent with these findings, ARS quantification displayed an enhancement of approximately 1.5-fold in calcification in LV-miR-148b-3p-transduced cells. Accordingly, ALP activity displayed about 2.5-fold increase in osteogenesis in LV-mir148b-3p-transduced human BM-MSCs after 14 days. Forced expression of this miR upregulated the osteoblast differentiation markers, ALP and ColI, as judged by immunostaining and Western blotting. Of note, our data confirmed that LVs could be a proper vehicle for effective delivery of miR to human BM-MSCs as demonstrated by high transduction efficiency.

MiR-148b belongs to miR-148/152 family, which consists of three members, miR-148a, miR-148b and miR-152. Members of this family are associated with growth and development of normal tissues and are expressed in different cells, particularly in stem cells. Moreover, they participate in regulating target genes, which are responsible for proliferation, differentiation and apoptosis. Expression of the members of this family is different in tumor and non-tumor diseases. Most studies have shown that members of this family act as an oncogene or suppressor of the tumor. Also, members of this family play an important role in non-tumor complications, such as type 1 diabetes and arthrosclerosis lesions. Moreover, miR-148a expression increases in multiple myeloma (MM), hepatocellular carcinoma (HCC) and medulloblastoma. Also, the expression of miR-148b is significantly increased in plasma of patients with breast cancer. Therefore, this group of micro-RNAs can be used as an important biomarker for the early diagnosis of these cancers [32]. Conversely, the expression of the members of this family in liver cancer stem cells, gastrointestinal cancers, cholangiocarcinoma, pancreatic duct adenocarcinoma, oral squamous cell carcinoma, ovarian cancer, endometrial adenocarcinoma and prostate cancer are reduced [32, 33].

Schoolmeesters et al demonstrated that miR-148b is increased in early osteogenic differentiation of human MSCs potentially via regulating various genes involved in skeletogenesis [10]. Consistent with our findings, recently it has been shown that mir-148b is expressed positively during osteogenic differentiation of MSCs derived from human amniotic fluid [34]. It has also been demonstrated that miR-148b downregulates noggin (NOG) by direct targeting [35]. NOG is an antagonist of bone morphogenetic proteins (BMPs) and inhibits their activities by binding to them [36] Concurrent with this notion, NOG negatively regulates BMP-induced osteblastogenesis [37] and bone development [38] . Consistently, Yoshizawa et al have elucidated the inhibition of NOG induced by osteogenic differentiation of human MSCs [39]. Other studies have shown that co-expression of miR-148b with BMP-2, promotes osteogenesis of human adipose-derived stem cells [40].

## Conclusions

The mechanism through which osteogenesis is increased in miR-148b-3p-transduced human BM-MSCs could be explained via direct targeting of NOG, but more research is needed to clarify the detailed molecular mechanisms. Based on promising findings of the current and other different studies, miR-148b-3p can be considered as a therapeutic agent in RNA-based therapy for skeletal disorders.

## Additional file

Additional file 1: **Figure S1.** Increased expression of miR-148b-3p in human BM-MSCs, which was mediated by LV enhanced osteogenic differentiation in T12.5 flasks after 21 days (DOC 432 kb)

## Data Availability

The data generated or analyzed during this study are included in this article

## Abbreviations

ALP: Alkaline phosphatase
ARS: Alizarin red S staining
BCIP/NBT: 5-bromo-4-chloro-3-indolyl phosphate/Nitrobluetetrazolium
BM-MSCs: bone marrow-derived mesenchymal stem cells
BMP: bone morphogenetic protein
ColI: collagen type I
DMEM-LG: Dulbecco’s modified Eagle’s medium-low glucose
HCC: multiple myeloma (MM), hepatocellular carcinoma
HRP: horseradish peroxidase
miRs: microRNAs
MSCs: Mesenchymal stem cells
PBS: phosphate-buffered saline
pNP: p-nitro-phenol
pNPP: p-nitro-phenyl phosphate
PVDF: polyvinylidene difluoride
RT: room temperature
SDS: sodium dodecyl sulfate
sh: short hairpain
TBS-T: Tris-buffered saline containing 5% BSA diluted in 0.1% Tween-20
