# Supplementary material for: Overexpression of MicroRNA-148b-3p stimulates osteogenesis of human bone marrow-derived mesenchymal stem cells: the role of MicroRNA-148b-3p in osteogenesis
Source: BMC Med Genet. 2019 Jul 1;20:117. doi: 10.1186/s12881-019-0854-3 (PMC6604430; doi:10.1186/s12881-019-0854-3)
Supplement: Supplementary file 1 — Figure S1. Increased expression of miR-148b-3p in human BM-MSCs, which was mediated by LV enhanced osteogenic differentiation in T12.5 flasks after 21 days (DOC 432 kb) [file 12881_2019_854_MOESM1_ESM.doc]

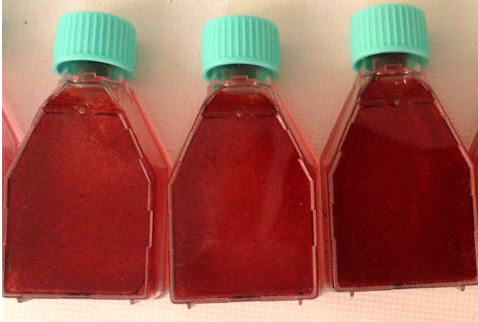


LV-Ctrl LV-148b-5p LV-148-3p

Supplementary Fig. 1 Increased expression of miR-148b-3p in human BM-MSCs, which was mediated by LV enhanced osteogenic differentiation in T12.5 flasks after 21 days.
